# Two Failures to Replicate High-Performance-Goal Priming Effects

**DOI:** 10.1371/journal.pone.0072467

**Published:** 2013-08-16

**Authors:** Christine R. Harris, Noriko Coburn, Doug Rohrer, Harold Pashler

**Affiliations:** 1 Psychology Department, University of California San Diego, La Jolla, California, United States of America; 2 Psychology Department, University of South Florida, Tampa, Florida, United States of America; Goldsmiths, University of London, United Kingdom

## Abstract

Bargh et al. (2001) reported two experiments in which people were exposed to words related to achievement (e.g., *strive, attain*) or to neutral words, and then performed a demanding cognitive task. Performance on the task was enhanced after exposure to the achievement related words. Bargh and colleagues concluded that better performance was due to the achievement words having activated a "high-performance goal". Because the paper has been cited well over 1100 times, an attempt to replicate its findings would seem warranted. Two direct replication attempts were performed. Results from the first experiment (n = 98) found no effect of priming, and the means were in the opposite direction from those reported by Bargh and colleagues. The second experiment followed up on the observation by Bargh et al. (2001) that high-performance-goal priming was enhanced by a 5-minute delay between priming and test. Adding such a delay, we still found no evidence for high-performance-goal priming (n = 66). These failures to replicate, along with other recent results, suggest that the literature on goal priming requires some skeptical scrutiny.

## Introduction

The term 'priming' is used to refer to a wide range of different changes in perception, judgment, and behavior that can be elicited by giving people a relatively minimal exposure to words, pictures, or other stimuli. Some types of priming effects are undoubtedly robust. Within the cognitive literature, one of the most widely investigated forms of priming is produced by having participants read a prime word and then try to classify a target letter string as a word or nonword. When the prime is related to the target string (e.g., when the prime is 'doctor' and the target is 'nurse') people respond more quickly [1]. Similarly, semantically related primes make people more accurate in naming briefly flashed target words [2]. These forms of perceptual priming effects (as well as others involving memory retrieval, e.g., McKoon & Ratcliff [3]) have been directly replicated many times. Such replications often have involved statistically powerful experiments, using within-subject comparisons with many trials collected for each participant in each of the experimental conditions.

### Perceptual versus Social/Goal Priming

The function and mechanism of the perceptual priming effects described in the previous paragraph seem relatively straightforward. Signal detection analysis shows that these priming effects reflect a perceptual bias toward assuming that target information is consistent with the prime [2,4,5], see 6 for discussion. From a Bayesian perspective, this type of bias mechanism may be a rational accommodation to a tendency for conceptually related things of all kinds to occur in close temporal proximity (cf. Huber, Shiffrin, Quach & Lyle [7]). A bias effect may arise by a simple mechanism of spreading activation in a network through which the representations of related things are highly connected (cf. Morton [8]).

Within the past 15 years or so, a more varied set of priming effects have been described in the literature. These effects involve changes in attitudes, performance level, choices of behavior, and motivational states--often apparently activating goals consonant with the content of the priming materials. For example, studies have reported effects such as reading words related to the elderly makes people walk more slowly as they exit the lab [9] while reading words related to money causes people to volunteer less of their time [10]. Other work has reported that seeing an American flag makes participants who are US residents report more politically conservative views when asked eight months later [11] and that plotting two closely spaced points on graph paper causes people to feel closer to their friends and family [12].

While it is common to refer to all the things described thus far as "priming", in fact there are some important differences between the social and goal priming studies just mentioned, and the perceptual priming effects discussed earlier. One difference is that whereas the perceptual priming effects have been directly replicated many times, direct replication attempts involving the social and goal priming literature appear to be relatively infrequent (cf. Yong [13]). Secondly, many of the direct replication attempts of which we are aware have failed to confirm social and goal priming effects (e.g. [14–17], for more general discussions, see 13,18). It is well known that positive results are far more likely to be published than negative results (the *file drawer problem* [19]). Thus, it is at least possible that the reported failures to replicate could conceivably reflect only the visible aspect of a more pervasive problem of replicability relating to goal priming. Third, whereas the function of perceptual priming seems easy to understand, as mentioned above, the functional purpose achieved by higher-level priming effects are less obvious (although see Dijksterhuis & Aarts [20], for a different perspective). If reading words is sufficient to activate concepts, and this automatically changes people’s selection of goals and actions, it would seem to open them up to potentially disadvantageous influences. Moreover, if the phenomenon is true, it would appear to have important practical implications, given the ease with which effects are purportedly achieved and in some cases, the reported long-term influence of these manipulations (e.g., Carter et al. [11]).

Finally, the difference between social/goal priming and perceptual priming that is perhaps most interesting is that--if judged from the published research alone--one would conclude that the effect size for social/goal priming as measured with Cohen’s d (which scales the effect against variability in the study population) may actually be *larger*. Pashler et al. [15] performed an unsystematic examination of some well-known goal priming studies and found effect sizes in the range of d=0.5 to d=1 (commonly labeled as large effects.) Effect sizes in the perceptual priming literature are harder to estimate from published data because the prime is manipulated within subject with repeated measures and eta-squared is the commonly reported effect size, which is not comparable to measures like Cohen’s d that scale effects against the variability between people in the population. However, Pashler et al. [15] reanalyzed perceptual priming data from Yap, Balota and Tan [21] and found an effect size of only d=.06. (This may or may not be representative of perceptual priming studies generally.) It is odd that social/goal priming effects would be stronger than perceptual priming effects, given the fact that the latter are normally assumed to reflect far more direct pathways (bias due to spreading activation; but see Ratcliff & McKoon [22]). Simmons, Nelson, and Simonsohn [23] have provided another reason for skepticism about the large effect sizes reported in the social/goal priming literature, pointing out that even quite "obvious" behavioral science effects (such as the tendency of people who like eggs to eat egg salad more often than other people) turn out, when properly measured, to be quite small (i.e., d < .5).

Thus, it seems at least possible that the social and goal priming literature might contain many large observed effects due to numerous false alarms. This could occur if a great number of small underpowered experiments have been conducted, with only those results reaching significance having been published [24,25].

The points raised above merely argue that some open-minded skepticism about social and goal priming effects may be in order--they obviously do not support any stronger conclusions than that. It is only by direct replication attempts that the validity of specific findings can be assessed. The present article contains the third in a series of direct replication attempts conducted in our lab looking at the replicability of influential social and goal priming results (other groups are engaged in similar efforts; see Bower [26].

The current paper attempts to replicate work from Bargh et al. [27] – a paper that has been extremely influential, having been cited well over a 1100 times according to Google Scholar, with more than 100 of these citations occurring just within 2012. Bargh and colleagues theorized that exposure to high achievement words would activate an unconscious goal to perform well in participants. In support of this hypothesis, they found that participants who were primed in such a way performed better on subsequent word search tasks. Here we report two attempts to directly replicate experiments from Bargh et al. [27]. In both experiments, we try to follow as closely as possible the methods and materials used in the original work, while also keeping experimenters blind to condition in order to be sure that results were not influenced by experimenter expectancy effects. (The purpose of the current paper was to examine whether the effects of unconscious high-performance-goal primes reported by Bargh and colleagues would replicate. Therefore, we focused our replication attempts on the first two experiments from Bargh et al. that directly pertain to this. Given our failure to find any hint of the purported priming effects, we did not attempt to replicate the additional experiments reported in Bargh et al.)

It should be noted that the large literature on social and goal priming effects contains other reports of different sorts of priming manipulations increasing achievement, in addition to the effect being examined in the present article (e.g., [28–30], see also Dijksterhuis & Aarts [20], for a general review).

## Experiment 1

Our first experiment focuses on Experiment 1 of Bargh et al. One group of their participants was primed with words that were predicted to activate an unconscious high-performance goal (i.e., they completed word-search puzzles that contained words such as compete, succeed, and win) while the control group completed puzzles with neutral words (e.g., ranch, carpet, and river). Both groups were then given an additional word search task. The high-performance-goal group located significantly more items on the subsequent word search puzzles than the control group. Seventy-eight participants were tested in Experiment 1 of Bargh et al. [27]; to provide greater statistical power, we tested 106 participants in the first replication attempt reported here.

### Method

We attempted to replicate the Bargh et al. Experiment 1 as directly as possible. We contacted John Bargh for his original stimuli; while Bargh responded in a helpful fashion to our request, he indicated that these materials were no longer available (except for a few fragmentary samples). Therefore, in cases where the exact stimulus specifications were not reported in the original paper (e.g., physical size of puzzle matrix), we used our best judgment in setting stimulus parameters, as specified below.

#### Participants and design

One hundred and six undergraduate students (73 females, 33 males) at the University of California, San Diego participated in the experiment for course credit. Participants were randomly assigned to either the high-performance-goal priming or neutral condition. The participants provided written informed consent. This research was approved by the University of California San Diego Human Research Protections Program.

#### Materials

The primes were embedded in a word search puzzle that consisted of a 10 x 10 matrix of letters, approximately 9.5 cm x 9.5 cm. The physical size of the matrix was not provided in the methods section of Bargh et al. [27], so we chose this size because it seemed convenient for participants. Thirteen target words were listed below the letter matrix, and the participant’s task was to find and circle all of the words on the list.

There were two conditions: high-performance-goal priming and neutral priming. All puzzles contained the same set of six neutral words (building, turtle, green, staple, lamp, plant). For participants in the high-performance-goal priming condition, the seven remaining words consisted of achievement related words (*win, compete, succeed, strive, attain, achieve, master*). For participants in the neutral priming condition, the seven words had no such connotations (these were: *ranch, carpet, river, shampoo, robin, hat, window*). The words in the letter matrix could appear vertically, horizontally, or diagonally; they could also run from right to left, left to right, bottom to top, or top to bottom. To ensure that the word search task was not inadvertently easier in one condition (e.g. the location or arrangement of the target words might help or hinder finding words), we had 5 puzzle matrix orders for each condition. The original Bargh et al. paper only used one matrix for the prime condition and a different matrix for the neutral condition, which were not available to the current authors.

The dependent variable also involved solving puzzles. Three test puzzles were used. Each puzzle was fashioned in a similar manner to those in the priming word-search task. However, in contrast to the priming word-search puzzle, no word list was provided below the test puzzles. Rather, one theme (*bugs, colors, or food*) was listed at the top of each puzzle, to indicate the category to which the hidden words belonged. The methods section of Bargh et al. only provided 5 of the 10 words used on each themed puzzle, therefore we created 5 additional words for each category. For the food theme, the words used were *cake, peach, eggs, corn, cabbage, bread, soup, bean, pasta* and *fish*. The bug theme contained the words, *roach, mosquito, beetle, moth, butterfly, wasp, spider, cricket, flea*, and *worm*. The color theme comprised the words, *red, purple, yellow, orange, tan, blue, white, brown, black*, and *green*. The dependent variable was the total number of words (out of 30) that the participant found in the three puzzles. The order of the test puzzles was counterbalanced.

#### Procedure

Careful steps were taken to ensure that the experimenter would remain blind to condition. Prior to the start of the experiment, an individual who was not otherwise involved in the study created the priming materials. He folded each puzzle in thirds and (lightly) taped it shut. Half of the puzzles contained the high-performance-goal prime and half contained the neutral prime. The stack of puzzles was then shuffled and a number from 1 to 120 was written on the outside of each form (so that the condition could be matched to the participant after the experiment). The puzzles were then placed in a cardboard box with an opening at the top that was large enough for participants to comfortably reach in and pull out a puzzle.

Up to two participants were run in each hour-long experimental session. They were greeted by the experimenter and instructed to turn off their cell phone in the waiting room. Participants were then escorted individually into separate private rooms and seated at a desk. After informed consent was obtained, participants were told that for their first task (which was the priming manipulation), they would need to select a paper from the cardboard box. The box was sitting next to the desk and contained all of the pre-randomized, taped, and numbered priming manipulation puzzles. The participants were instructed to reach into the box and select their sealed paper to prevent the experimenter from handling any of the forms and inadvertently become aware of the condition. Participants were instructed not to open or show the folded puzzle to the experimenter, but to state the number located on the outside of the paper. This number would later be used to match up the priming condition with the test puzzles at the end of the experiment. For the priming manipulation task, participants were told that once the experimenter left the room, they could open their puzzle and take as long as they needed to complete the task in private. The original Bargh et al. study stated that upon completion of the priming puzzle, the experimenter asked participants to put that puzzle off to the side. We asked participants to put it face down in a bin to help ensure that the experimenter would remain blind to condition. Participants then pressed a button (similar to a doorbell) sitting on their desk to let the experimenter know they were ready for the next task. Bargh et al. did not state the location of the experimenter during the priming manipulation or indicate how the experimenter knew when the participant had completed their priming task (e.g. Experimenter stayed in room during the priming manipulation task, Experimenter waited outside of the room.)

Our experimenter waited outside the participant’s room until s/he heard the sound of the bell. The chime for the bell was located outside of the participant’s room to prevent any additional distraction. The experimenter opened the door after hearing the bell, but remained in the doorway (roughly 10ft away) until the participant confirmed that the priming puzzle was sitting face down. Then the experimenter re-entered the room to provide participants with the three dependent variable puzzles. The experimenter explained to participants that they would now complete three more word search puzzles and that this time there was a theme listed at the top of each puzzle. The participants’ task was to find as many words as they could that matched the theme. As in the Bargh et al. experiment, participants were told to work on the puzzles in any order they wished, going back and forth between them if they chose to do so. They were informed that they had 10 minutes to complete all three puzzles. At the end of the 10-minute period, the experimenter announced that time was up, reminded participants not to write their names on the paperwork, and asked the participant to place all four puzzles into a sealed box provided for the purpose. Participants then completed a demographics questionnaire.

Upon completion of the experiment, a "funnel debriefing" questionnaire was used to probe any awareness or suspicions the participant might have about the priming manipulation. The questionnaire began with questions designed to ascertain whether the participant understood the instructions. Participants were then asked what they thought the experiment was about, whether they felt that one task in the experiment may have affected another task, and to try to guess how the first word puzzle could have been related to the later puzzle tasks [27]. Finally, participants read over a debrief sheet explaining the purpose of the experiment, at which point they were thanked for their participation.

### Results and Discussion

Eight participants were excluded from the analyses. Three figured out the hypothesis (that the achievement words were related to performance on the subsequent tasks), and one indicated at the end of the session that he had already previously participated. Two participants experienced technical problems (specifically, the button for the bell did not work which resulted in more time between the priming puzzle and test puzzles), and two circled all but one of the target words on the priming puzzle. (While these last four participants were excluded, their inclusion did not alter the pattern of results.) Thus, in the final analyses, there were 98 participants (48 in the high-performance-goal priming condition and 50 in the neutral condition).

Participants sometimes circled words that were thematically correct but comprised only part of the longer target word. For example, the target word “PEACH” contains a shorter unintended target (i.e., PEA), both of which correctly fall under the FOOD category. This occurred 62 times. All 62 of the cases of unintended targets were from four words used in Bargh et al. [27]: BUTTERFLY (15 participants circled FLY), BEETLE (14 participants circled BEE), PEACH (25 participants circled PEA), and EGGS (8 participants circled EGG). (Bargh et al. make no mention of how they dealt with this issue, although presumably it would have arisen in their study as well given that the same stimuli were used.) 

The data were analyzed using two scoring systems. In the stringent scoring system, points were given only when the full intended target words were circled. In the more lenient scoring system, points were given for shorter words that overlapped the target words, as long as they technically qualified as belonging to the theme of the puzzle. For example, a point was given if the intended target was missed (e.g. BUTTERFLY), but the unintended overlapping target was located (e.g. FLY). However, if a participant circled both the unintended overlapping target (e.g. FLY) and the intended target word (BUTTERFLY) only one point was issued, not two. It was never the case that participants found an unintended target word that was not a subcomponent of the intended target (e.g., FLY and BUTTERFLY positioned in two separate locations on the word search puzzle).

**Figure 1:**
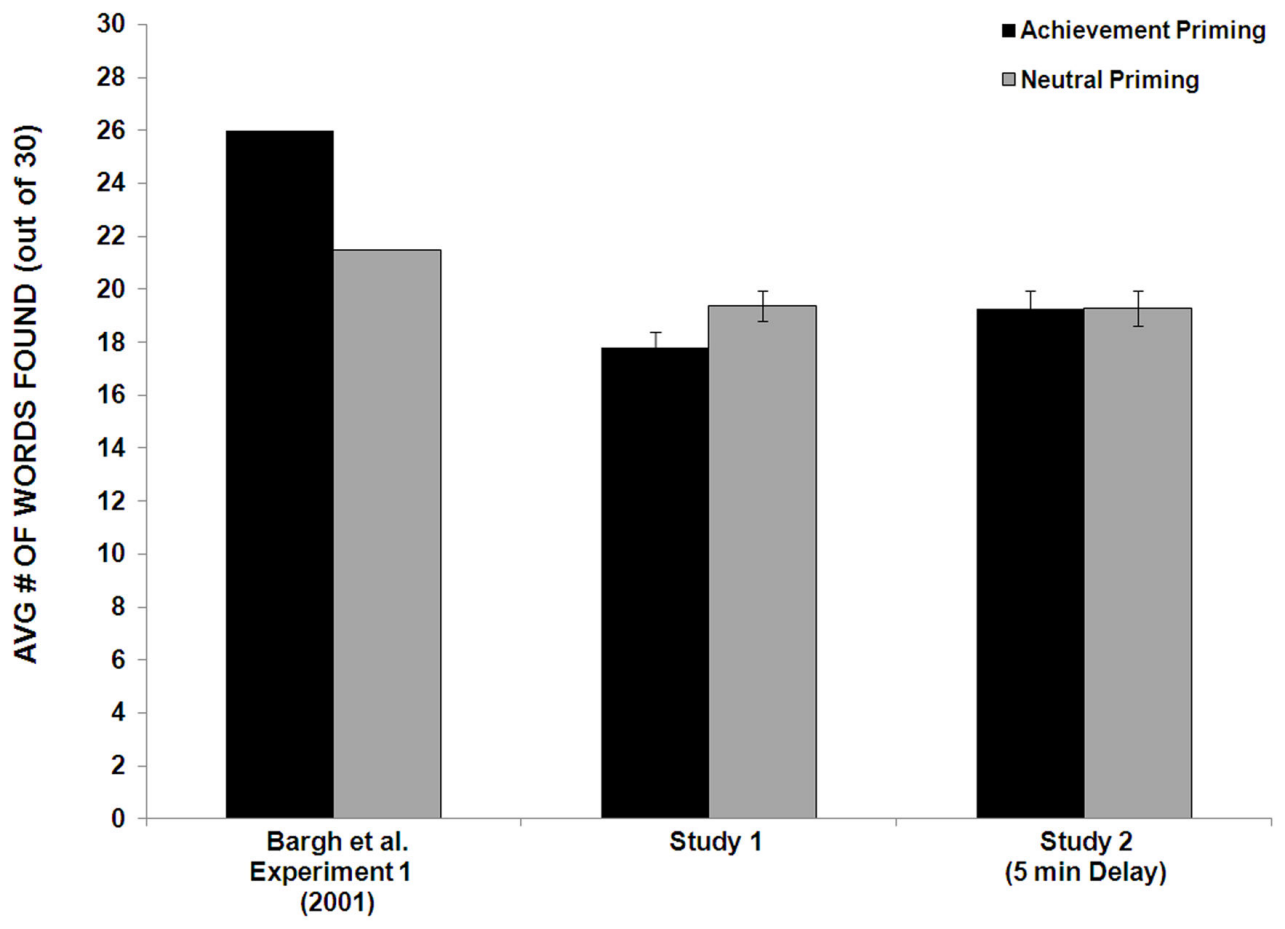
Mean number of words found across all test puzzles (+SE), as a function of priming condition (high-performance-goal vs. neutral), for Experiment 1 in Bargh et al. (2001), Experiment 1, and Experiment 2. (SE was not reported in Bargh et al., 2001).

Our results using a stringent scoring system (where the score counts only intended target words) are shown in Figure 1 along with the results for Experiment 1 in Bargh et al. We performed a between-subjects ANOVA with condition and gender as factors. (Gender was analyzed in the original Bargh et al. paper so we included it here.) In contrast to Bargh et al., there was no effect of prime condition on subsequent performance *F*(1,94) = 1.45, p = .23. This corresponds to an effect size of d = -0.24 (95% confidence interval ranging from 0.15 to -0.64).

In fact, if anything, participants tended towards locating *fewer* words in the high-performance-goal priming condition (M = 17.81, SD = 3.97) than in the neutral condition (M = 19.36, SD = 3.58). There was also no main effect of gender, *F*(1,94) = .038, p = .85, nor an interaction of gender with condition, *F*(1,94) = 2.92, p = .09. (Bargh et al. also did not find a significant effect of gender or an interaction with gender.) 

The results also were scored using a more lenient scoring system, giving credit if the intended target was missed, but a shorter overlapping target word was found that was consistent with the theme of the puzzle. Again, the results showed no main effect for priming condition (nor were the means in the correct direction): performance with high-performance-goal priming (M = 18.48, SD = 3.93) vs. with neutral priming (M = 19.96, SD = 3.53), *F*(1,94) = 1.36, p = .25, d=-0.24, CI ranging from +0.16 to -0.63). There was no effect of gender, *F*(1,94) = .11, p = .74, nor a significant interaction of gender and prime, *F*(1,94) = 2.54, p = .11.

A reviewer asked how outliers might have impacted the findings. As a subsidiary analysis, we compared medians for the stringent scored data; these were 18 for the high performance priming condition and 20 for the neutral priming condition.

In sum, this experiment attempted to directly replicate the high-performance-goal priming results reported in Experiment 1 of Bargh et al. We found no evidence that achievement word primes led to enhanced performance, nor were the means in the correct direction for such an effect.

## Experiment 2

Bargh et al. [27] reported another experiment that specifically focused on nonconscious high-performance-goal priming. In Experiment 3, Bargh et al. reasoned that if their effects from Experiment 1 were due to unconscious goal-priming (as opposed to other possible mechanisms such as construing the task differently or priming of a particular behavior), then the effects of the goal-prime should get even stronger over time. The authors write, “one important general quality of goal pursuit is that goal strength increases, rather than decreases, over time until the goal is attained.” (p. 1016). Therefore, in their Experiment 3, Bargh et al. used the same measures and methods used in their first experiment, but included an additional manipulation of delay between the priming puzzle and the final measure of performance. After completing the high-performance-goal prime, some of the participants were given a 5-minute filler task to perform before doing the final word search tasks. Bargh et al. found that this more than doubled the size of the priming effect.

Given that our first experiment was unable to replicate the high-performance-goal priming effect reported by Bargh et al., it seemed that a useful follow-up would be to examine whether their apparently more powerful delay condition could reveal an effect of goal priming in a replication study. Thus, in the next experiment, we attempt a direct replication of the Bargh et al. effect for their delayed goal-priming condition using their procedures and materials to the extent possible (Given our interests, we did not include the additional conditions in Bargh et al. that did not involve goal priming, e.g., impression formation).

### Method

#### Participants

Seventy-two undergraduate students (49 females, 23 males) at the University of California, San Diego participated in the experiment for course credit. The participants provided written informed consent, and the research was approved by the University of California San Diego Human Research Protections Program.

#### Materials

The priming manipulation and test puzzles were the same as in Experiment 1, but the administration of these two were separated by a 5-minute delay. Following Bargh et al. [27], this delay period involved having participants draw their family tree with as much detail as possible. Participants were told to begin by placing their immediate family (father, mother, brothers, sisters) at the bottom of a blank 8.5 x 11 inch sheet of paper provided to them. Then, using a branch diagram, they were to continue back one generation at a time and include aunts, uncles, and cousins.

#### Procedure

The procedures were the same as in Experiment 1, with the exception of the additional 5-min delay task. Before beginning the priming puzzles, participants were told they would be doing three unrelated tasks (as stated in Bargh et al.). They then performed the priming task (high-performance-goal vs. neutral). Afterwards, they were given the delay task (i.e. completing the family tree diagram). After 5 minutes, the experimenter entered the room, asked them to stop working on the tree diagram, and to place the paper face down in the adjacent bin. Participants then completed the three word-search test puzzles.

### Results and Discussion

Six participants were discarded from the analyses, leaving 32 participants in the high-performance-goal priming condition and 34 in the neutral condition. One participant figured out the manipulation (revealed at debriefing). Another forgot to push the button to signal the experimenter to enter the room upon completion of the priming puzzle. One participant circled every individual letter for each of the 13 target words on the priming puzzle, disrupting the cohesive appearance of the word. Three participants finished their family tree early and pushed a button before the end of their 5 min delay period, resulting in extended interaction with the experimenter, possibly disrupting the experiment. The pattern of results is the same regardless of whether the five participants (excluding the participant who figured out the manipulation) are included in the analyses.

There were 31 occurrences of participants finding unintended overlapping target words: BUTTERFLY (12 participants circled FLY), BEETLE (6 circled BEE), and PEACH (13 circled PEA). The same stringent and lenient scoring systems used in Experiment 1 were applied here.

We employed the same analysis strategy that we used in the last experiment -- a between-subjects ANOVA with condition and gender as factors. (Bargh and colleagues performed a full ANOVA that included all of their conditions, followed by specific contrasts, but did not provide the key contrast comparing the high-performance-goal priming group to neutral priming group at the 5 min delay.) 

Using our stringent scoring system, the means for the two conditions were virtually identical: high-performance-goal priming condition (M = 19.28, SD = 3.72, median = 19.5) vs. neutral priming condition (M = 19.29, SD = 4.06, median = 19). There was no hint of an effect of prime condition on subsequent performance after a five-minute delay, *F*(1,62) = 0.02, p=.88. The effect size for priming was -0.03, with a 95% confidence interval ranging from 0.45 to -.52.

Figure 1 provides the means (+SE). There was a significant main effect of gender, *F*(1,62) = 4.79, p = .03, with females locating more words (M = 20.00, SD = 3.69) on the test puzzles than did males (M = 17.76, SD = 3.87). There was no interaction of gender with condition, *F*(1,62) = 1.08, p =.30.

The ANOVA of lenient scoring revealed the same pattern of results. There was no effect of the priming manipulation: neutral priming (M=19.66, SD = 3.69) vs. high-performance-goal priming (M = 19.85, SD = 3.77), *F*(1,62) = 0.10, p=.75. The effect size for priming was -0.08, with a 95% confidence interval ranging from 0.41 to -.56. The effect of gender was again significant, *F*(1,62) = 4.74, p = .03. Females located M = 20.44 (SD = 3.52) while males located M = 18.29 (SD = 3.76). The effect of gender did not interact with condition, *F*(1,62) = 0.96, p = .33.

## General Discussion

The present work included two direct attempts to replicate the "high-performance-goal priming effect" reported by Bargh et al. [27]. Neither experiment confirmed the original results.

As noted above, our experiments were designed to ensure that the experimenter could not have known what condition the participant was in and therefore could not have produced an artifactual priming effect. Bargh et al. [27] indicate also taking some precautions against such artifacts. Thus, it seems likely that experimenter expectancy effects in the original study are not the reason for the difference in outcomes.

Another obvious possibility is that the high-performance-goal prime results of Bargh et al. [27] are invalid, and may be due, for example, to Type 1 errors. The possible rate of such errors in the goal-priming literature is based on speculation and is currently quite controversial [26,31,32]. The published literature can provide an inaccurate picture of the entirety of the research that has been conducted on a topic due to the "file drawer problem" -- the strong inclination for scientific journals to selectively publish positive findings and their disinclination to publish failures to replicate and null results (cf. Rosenthal [19], see also Simmons, Nelson & Simonsohn [33]). The disinclination to publish replication failures is increasingly recognized as harmful to the credibility of many scientific fields [24,34–36].

There are also technical aspects of the Bargh et al. [27] design that could have potentially allowed the introduction of very subtle confounds. As noted above, the priming manipulation was embedded in a word search task, and the final outcome measure also involved a word search task. It cannot be assumed that any effects of solving a particular priming word task upon performance of a later word task must necessarily be mediated by priming (i.e., depending solely upon the identity of the word that was sought or discovered.) It is also conceivable that certain puzzles promoted learning of skills or tendencies that would prove more or less useful for particular test puzzles. As noted above, Bargh et al. [27] evidently used just a single priming puzzle for each condition. If there was more positive transfer from one puzzle than another, their design could misconstrue this as a priming effect. To take a hypothetical example, suppose the achievement priming puzzles included a disproportionate number of words running along a particular diagonal, and the test puzzles had a similar characteristic; practicing the former might specifically enhance learning of the latter.

Additionally, we noticed inconsistencies in the data graphed in Figure 1 of Bargh et al. [27] and wondered if the difference between our results and those of Bargh et al. might be due to errors in their analyses. Specifically, the z-scores presented in that figure do not average to zero as they should. We corresponded with Bargh (personal communication, March 1, 2013), who acknowledged that there were erroneous numbers presented in the right-side of the figure but stated that these errors were not in the analysis. Thus, it would appear that this does not account for the differences between our findings.

Finally, the experimenter was not in the room with the participant in our experiments, which might have differed from the original experiments, and might for some reason be important (this possibility was suggested by John Bargh in a review of the current article). However, goal priming studies that use Internet presentations are appearing in the literature (e.g., Caruso, Vohs, Baxter & Waytz [37]); thus, reported priming findings apparently can occur without experimenter presence.

### Statistical Considerations

One can never completely rule out the possibility that even multiple failures to replicate a finding merely reflect sampling error in the replication attempts ("Type 2 error"). However, several analyses may shed some light on the likelihood of this possibility.

A natural place to start is with the effect sizes in the original study. Again, the basic finding we sought to replicate was better performance on the word search task for participants assigned to the high-performance-goal priming condition, as reported in Experiments 1 and 3 of Bargh et al. [27]. In their Experiment 1, the priming effect on word search was tested with an ANOVA, which yielded a value of *F*(1,74)= 9.64. Assuming equal ns in the two priming conditions (their paper does not state the ns for each condition), we estimate that the observed effect size is .70 (calculated using the Campbellcollaboration.org tool). To determine the most relevant effect size in Bargh et al. Experiment 3 (i.e., for the priming effect with a 5-minute delay as examined in our Experiment 2), we would need the t or F value for the contrast between the priming and neutral condition for the 5-minute delay condition (their experiment also included a zero-delay condition). Unfortunately, this was not reported. However, the paper did report a significant difference, p < .04, for the effect of priming at zero delay, and it also reported a significant priming X delay interaction, p < .01, reflecting greater priming effect at 5-minute delay as compared to zero delay. Thus, there is no doubt of statistical significance, but the effect size cannot be determined nor (as far as we can tell) reliably estimated from the data provided.

We used the effect size available from Experiment 1 in the original Bargh et al. paper, to assess how much power the present experiments have to detect such an effect. Experiment 1 had 98 valid subjects, which affords a power of .93 to detect an effect of d = .70 (according to G*Power [38];, 2-tailed test). Experiment 2 had 66 subjects, which corresponds to a power of .80 (2-tailed). So for the reported effect size of Experiment 1 of Bargh et al. [27], the power seems excellent. (Given that the 5-min delay condition used in Bargh and colleagues’ Experiment 3 was significantly stronger than their no delay condition, one might presume that the effect size for this study was probably larger than that of Experiment 1, and thus this estimate seems conservative.) 

Of course, it is also possible that there is a true (nonzero) effect but the effect is much smaller than d=.70. If that were the case, the power of the present experiments to detect such an effect would be another matter. For example, if the true effect were d = .20, neither of our experiments would have even a 20% chance of detecting the effect. (It should be noted that the original Experiment 1 of Bargh et al. would have been even more unlikely to detect such an effect. Moreover, their results would not even reliably distinguish such a hypothetical effect from a similarly weak effect in the opposite direction.)

One can also view this situation from a meta-analytic perspective, and derive a synthetic mean effect size with its confidence interval. This amounts to assuming that all the observed effects are noisy estimates of the true effect size. (While this approach is often recommended by statistically sophisticated writers, the resulting synthesis is not necessarily accurate when data are biased, e.g., due to publication bias or other forms of nonrandom error.) In any case, if we combine the effect size from Bargh et al. [27] Experiment 1 (d = .70), and the corresponding effect size estimate from the current Experiment 1 (d = -0.24), the synthetic mean is d = .16, with a confidence interval ranging from -0.14 to 0.46. (Unfortunately, given the lack of effect size for Bargh et al. Experiment 3, we cannot combine that with the effect of the current Experiment 2, nor can we compute the synthetic mean of all four studies.)

So where does this leave us? We think that a fair summary of the situation would be: If the effect size were real and of a magnitude similar to the effects reported by Bargh et al. [27] (Experiment 1), then we would very likely have found a significant effect in both of our experiments.

## Conclusions

The phenomenon of high-performance-goal priming appears at first glance to be a very interesting discovery with potential practical applications (especially given its apparent large effect size, based on the original studies). Unfortunately, the current results do not confirm the reality of these effects. Interestingly, Shanks et al. [17] recently presented a series of nine studies examining a closely related form of priming reported by Dijksterhuis and van Knippenburg [39] whereby people are led to perform better on a general knowledge task after being primed by thinking about the stereotype of the college professor. Using a total of 475 participants; Shanks and colleagues were unable to reproduce the priming phenomenon. (See also Elder, Leipert, Musch, & Klauer [40] for another failure to replicate that result.) 

Additional research on the reproducibility of goal priming in general (beyond just the case of high-performance-goal priming) is clearly in order, and it is to be hoped that many investigators will attempt to closely repeat studies from some of the now-in-doubt literature (see also 13,18). Recognition of the importance of direct replication seems to be rapidly growing. It has been common to suppose that "conceptual replications" (in which many elements of a study are deliberately changed) may be an adequate substitute for direct replications. Unfortunately, however, this substitution may exacerbate the problem of publication bias. Pashler and Harris [25] argued that when conceptual replications succeed, they have a high likelihood of being published, whereas when they fail, they probably do not result in even so much as private skepticism of the original result [25].

Findings from the priming area have drawn an unusual amount of public attention, due to their surprising nature, apparently inspiring a great many conceptual replications. If the effects are real, the methods by which they can be repeated need to be clarified. If the effects are not real, the field needs to better understand how errors could enter the literature in such disturbingly large numbers, and how this kind of problem can be averted in the futures, for the benefit of science and for the reputation of experimental psychology as a discipline.

## References

[1] Meyer DE, Schvaneveldt RW (1971) Facilitation in recognizing pairs of words: Evidence of a dependence between retrieval operations. J Exp Psychol Hum Learn 90: 227-234. 10.1037/h0031564 5134329

[2] Schvaneveldt RW, McDonald JE (1981) Semantic context and the encoding of words: Evidence for two modes of stimulus analysis. J Exp Psychol Hum Percept Perform 7: 673-687. doi:10.1037/0096-1523.7.3.673.

[3] McKoon G, Ratcliff R (1979) Priming in episodic and semantic memory. J Verbal Learning Verbal Behav 18: 463-480.

[4] Johnston JC, Hale BL (1984) The influence of prior context on word identification: Bias and sensitivity effects. Attention & Performance. X: 243-255.

[5] Ratcliff R, McKoon G, Verwoerd M (1989) A bias interpretation of facilitation in perceptual identification. J Exp Psychol Learn Mem Cogn 15: 378-387. doi:10.1037/0278-7393.15.3.378. PubMed: 2524543. 2524543 10.1037//0278-7393.15.3.378

[6] Pashler H (1999) The psychology of attention. Massachusetts: MIT Press.

[7] Huber DE, Shiffrin RM, Quach R, Lyle KB (2002) Mechanisms of source confusion and discounting in short-term priming: 1. Effects of prime duration and prime recognition. Mem Cogn 30: 745-757. doi:10.3758/BF03196430. 10.3758/bf03196430 12219891

[8] Morton J (1969) Interaction of information in word recognition. Psychol Rev 76: 165-178. doi:10.1037/h0027366.

[9] Bargh JA, Chen M, Burrows L (1996) Automaticity of social behavior: Direct effects of trait construct and stereotype activation on action. J Pers Soc Psychol 71: 230-244. doi:10.1037/0022-3514.71.2.230. PubMed: 8765481. 8765481 10.1037//0022-3514.71.2.230

[10] Vohs KD, Mead NL, Goode MR (2006) The psychological consequences of money. Science 314: 1154–1156. doi:10.1126/science.1132491. PubMed: 17110581. 17110581 10.1126/science.1132491

[11] Carter TJ, Ferguson MJ, Hassin RR (2011) A single exposure to the American Flag shifts support toward Republicanism up to 8 months later. Psychol Sci 22: 1011-1018. doi:10.1177/0956797611414726. PubMed: 21742933. 21742933 10.1177/0956797611414726

[12] Williams LE, Bargh JA (2008) Keeping one’s distance: The influence of spatial distance cues on affect and evaluation. Psychol Sci 19: 302-308. doi:10.1111/j.1467-9280.2008.02084.x. PubMed: 18315805. 18315805 10.1111/j.1467-9280.2008.02084.x PMC2394280

[13] Yong E (2012) A failed replication attempt draws a scathing personal attack from a psychology professor. Discover Magazine blog. Available: http://blogs.discovermagazine.com/notrocketscience/2012/03/10/failed-replication-bargh-psychology-study-doyen/. Accessed 2012 March 22.

[14] Doyen S, Klein O, Pichon C-L, Cleeremans A (2012) Behavioral Priming: It’s All in the Mind, but Whose Mind? PLOS ONE 7: e29081. doi:10.1371/journal.pone.0029081. PubMed: 22279526. 22279526 10.1371/journal.pone.0029081 PMC3261136

[15] Pashler H, Coburn N, Harris CR (2012) Priming of social distance? Failure to replicate effects on social and food judgments. PLOS ONE 7: e42510. doi:10.1371/journal.pone.0042510. PubMed: 22952597. 22952597 10.1371/journal.pone.0042510 PMC3430642

[16] Pashler H, Rohrer D, Harris CR (2013) Can the goal of honesty be primed? J Exp Soc Psychol 49: 959-964. doi:10.1016/j.jesp.2013.05.011.

[17] Shanks DR, Newell BR, Lee EH, Balakrishnan D, Ekelund L et al. (2013) Priming intelligent behavior: An elusive phenomenon. PLOS ONE 8: e56515. doi:10.1371/journal.pone.0056515. PubMed: 23637732. 23637732 10.1371/journal.pone.0056515 PMC3634790

[18] Bartlett T (2012) Is psychology about to come undone? Chronicle of Higher Education. Accessed 2012, April. p. 17

[19] Rosenthal R (1979) An introduction to the file drawer problem. Psychol Bull 86: 638-641. doi:10.1037/0033-2909.86.3.638.

[20] Dijksterhuis A, Aarts H (2010) Goals, attention and (un)consciousness. Annu Rev Psychol 61: 467-490. doi:10.1146/annurev.psych.093008.100445. PubMed: 19566422. 19566422 10.1146/annurev.psych.093008.100445

[21] Yap MJ, Balota DC, Tan SE (2013) Additive and interactive effects in semantic priming: Isolating lexical and decision processes in the lexical decision task. J Exp Psychol Learn Mem Cogn 39: 140–158. doi:10.1037/a0028520. PubMed: 22612169. 22612169 10.1037/a0028520

[22] Ratcliff R, McKoon G (1988) A retrieval theory of priming in memory. Psychol Rev 95: 385-408. doi:10.1037/0033-295X.95.3.385. PubMed: 3406246. 3406246 10.1037/0033-295x.95.3.385

[23] Simmons JP, Nelson LD, Simonsohn U (2013) Life after p-hacking. Meeting of the Society for Personality and Social Psychology. New Orleans, LA 17-19 January 2013 Available: http://papers.ssrn.com/sol3/papers.cfm?abstract_id=2205186.

[24] Ioannidis JPA (2005) Why most published findings are false. PLOS Med 2: e124. doi:10.1371/journal.pmed.0020124. PubMed: 16060722. 16060722 10.1371/journal.pmed.0020124 PMC1182327

[25] Pashler H, Harris C (2012) Is the replicability crisis overblown? Three arguments examined. Perspect Psychol Sci 7: 531-536. doi:10.1177/1745691612463401. 26168109 10.1177/1745691612463401

[26] Bower B (2012) The hot and the cold of priming: Psychologists are divided on whether unnoticed cues can influence behavior. Science News 181: 26-29. PubMed: 22609116

[27] Bargh JA, Gollwitzer PM, Lee-Chai A, Barndollar K, Trötschel R (2001) The automated will: Nonconscious activation and pursuit of behavioral goals. J Pers Soc Psychol 81: 1014-1027. doi:10.1037/0022-3514.81.6.1014. PubMed: 11761304. 11761304 PMC3005626

[28] Custers R, Aarts H, Oikawa M, Elliot A (2009) The nonconscious road to perceptions of performance: Achievement priming augments outcome expectancies and experienced self-agency. J Exp Soc Psychol 45: 1200-1208. doi:10.1016/j.jesp.2009.07.013.

[29] Shantz A, Latham GP (2009) An exploratory field experiment of the effect of subconscious and conscious goals on employee performance. Organ Behav Hum Decis Processes 109: 9-17. doi:10.1016/j.obhdp.2009.01.001.

[30] Shantz A, Latham GP (2011) The effect of primed goals on employee performance: Implications for human resource management. Hum Resour Manage 50: 1-11. doi:10.1002/hrm.20409.

[31] Bargh JA (2012) The natural unconscious: Automaticity in cognition, motivation, and emotion.Psychology Today blog. Available: http://www.psychologytoday.com/blog/the-natural-unconscious/201203/nothing-in-their-heads Accessed 2012, May 7

[32] Bargh JA (2012) Priming effects replicate just fine, thanks. Psychology Today blog. Available: http://www.psychologytoday.com/blog/the-natural-unconscious/201205/priming-effects-replicate-just-fine-thanks Accessed 2012, May 11

[33] Simmons JP, Nelson LD, Simonsohn U (2011) False-positive psychology: Undisclosed flexibility in data collection and analysis allows presenting anything as significant. Psychol Sci 22: 1359-1366. doi:10.1177/0956797611417632. PubMed: 22006061. 22006061 10.1177/0956797611417632

[34] Funder D (2012) Replication, period. The Hardest Science. Available: http://hardsci.wordpress.com/2012/09/21/replication-period-a-guest-post-by-david-funder/ Accessed 2012, September 21.

[35] Pautasso M (2010) Worsening file-drawer problem in the abstracts of natural, medical and social science databases. Scientometrics. doi:10.1007/s11192-010-0233-5.

[36] Young NS, Ioannidis JPA, Al-Ubaydli O (2008) Why current publication practices may distort science. PLOS Med 5: 1418-1422. PubMed: 18844432. 10.1371/journal.pmed.0050201 PMC2561077 18844432

[37] Caruso EM, Vohs KD, Baxter B, Waytz A (2013) Mere exposure to money increases endorsement of free-market systems and social inequality. J Exp Psychol Gen 142: 301-306. doi:10.1037/a0029288. PubMed: 22774789. 22774789 10.1037/a0029288

[38] Buchner A, Erdfelder E, Faul F, Lang A (2009) G*Power (Version 3.1.2) [Computer program]. Available: http://www.psycho.uni-duesseldorf.de/aap/projects/gpower/.

[39] Dijksterhuis A, van Knippenberg A (1998) The relation between perception and behavior, or how to win a game of Trivial Pursuit. J Pers Soc Psychol 74: 865-877. doi:10.1037/0022-3514.74.4.865. PubMed: 9569649. 9569649 10.1037//0022-3514.74.4.865

[40] Eder A, Leipert C, Musch J, Klauer K-C (2012) Failed replication to prime intelligent behavior. Available: http://www.PsychFileDrawer.org/replication.php?attempt=MTI0 Accessed 2012, October 07.

